# Informal “Seed” Systems and the Management of Gene Flow in Traditional Agroecosystems: The Case of Cassava in Cauca, Colombia

**DOI:** 10.1371/journal.pone.0029067

**Published:** 2011-12-12

**Authors:** George A. Dyer, Carolina González, Diana Carolina Lopera

**Affiliations:** 1 The James Hutton Institute, Aberdeen, United Kingdom; 2 LACBiosafety Project, International Center for Tropical Agriculture (CIAT), Cali, Colombia; 3 International Food Policy Research Institute (IFPRI), Washington, D.C., United States of America; 4 LACBiosafety Project, International Center for Tropical Agriculture (CIAT), Cali, Colombia; United States Department of Agriculture, United States of America

## Abstract

Our ability to manage gene flow within traditional agroecosystems and their repercussions requires understanding the biology of crops, including farming practices' role in crop ecology. That these practices' effects on crop population genetics have not been quantified bespeaks lack of an appropriate analytical framework. We use a model that construes seed-management practices as part of a crop's demography to describe the dynamics of cassava (*Manihot esculenta* Crantz) in Cauca, Colombia. We quantify several management practices for cassava—the first estimates of their kind for a vegetatively-propagated crop—describe their demographic repercussions, and compare them to those of maize, a sexually-reproduced grain crop. We discuss the implications for gene flow, the conservation of cassava diversity, and the biosafety of vegetatively-propagated crops in centers of diversity. Cassava populations are surprisingly open and dynamic: farmers exchange germplasm across localities, particularly improved varieties, and distribute it among neighbors at extremely high rates vis-à-vis maize. This implies that a large portion of cassava populations consists of non-local germplasm, often grown in mixed stands with local varieties. Gene flow from this germplasm into local seed banks and gene pools via pollen has been documented, but its extent remains uncertain. In sum, cassava's biology and vegetative propagation might facilitate pre-release confinement of genetically-modified varieties, as expected, but simultaneously contribute to their diffusion across traditional agroecosystems if released. Genetically-modified cassava is unlikely to displace landraces or compromise their diversity; but rapid diffusion of improved germplasm and subsequent incorporation into cassava landraces, seed banks or wild populations could obstruct the tracking and eradication of deleterious transgenes. Attempts to regulate traditional farming practices to reduce the risks could compromise cassava populations' adaptive potential and ultimately prove ineffectual.

## Introduction

The applicability of ecological concepts and methods to environmental management is perhaps nowhere as clear as in agroecosystems, particularly in centers of crop origin and diversity, where farmers' management of seed is an integral part of the ecology of crops and their wild relatives [1], [2]. Crop landraces have been described as managed populations—open and dynamic systems that evolve in response to gene flow and selection [3], [4]. Exchange of planting materials among farmers is considered a major selective pressure and partly responsible for these populations' diversity [5], [6]. Scientists also recognize that seed exchange made domestication a more complex process than once thought. Yet, the complexity of farmers' past and present role in crop evolution is not fully appreciated. On one hand, farmer management does not reduce to seed exchange; cassava farmers, for instance, exercise frequency-dependent selection when conserving rare seed [5] and selection for heterozygosity when protecting volunteer seedlings [7]. But neither does management reduce to a selection pressure. In fact, seed exchange often is regarded as a random force, more akin to genetic drift than to selection [8], [9]. More generally, seed management and other farming practices constitute a set of forces that are intrinsic to a crop's demography and thus have quantifiable effects on gene flow and frequencies [9], [10]. That these effects have not been quantified bespeaks lack of an appropriate analytical framework.

Seed exchange can have unintended consequences, including the propagation of crop diseases. Arguably, it was also the main mechanism for the spread of biological innovations during the onset of farming—a role that it continues to play in developing areas to date [10]. Agricultural innovations more generally have influenced every aspect of farming throughout history, from input use to land-use patterns. Innovations embodied in the seed, including genes of agronomic value and more recently transgenes, also entail *sui generis* risks [11]. Although studied extensively in industrialized countries, the unintended implications of biotechnology cannot yet be fully ascertained in centers of crop diversity, where transgenes could introgress into landraces and their wild relatives [12]. This applies to vegetatively-propagated crops, e.g., cassava and potato, which have been largely absent from discussions on biosafety [1], [13]. Managing gene flow and other repercussions of farmer practices requires unraveling these practices' intimate association with crop demography.

In this paper we use a demographic model that construes seed-management practices as events in the life history of crops to describe the dynamics of cassava (*Manihot esculenta* Crantz). We estimate various demographic parameters for cassava in Cauca, Colombia—the first estimates of their kind for a vegetatively-propagated crop—and compare cassava's population dynamics with those of maize, a sexually-reproduced grain crop. We discuss the implications for gene flow, the conservation of cassava diversity, and the biosafety of vegetatively-propagated crops in centers of diversity.

### Analytical framework

Cassava is a perennial shrub whose starchy, tuberous roots are a major source of carbohydrates in tropical countries. Numerous landraces of this crop are maintained in farming communities throughout the Amazon basin's rim, including Colombia [5], [14]. In the wild, the species' diversity is highest in south-western Brazil, cassava's center of origin. Clonal propagation has not isolated cassava reproductively, so its genes (and transgenes) can introgress into wild *Manihot* wherever their distributions overlap [1]. Wild *M. esculenta* is absent in Colombia, but four other potentially intercrossing *Manihot* species are present [14]. A possible containment strategy thus could require restricting commercial release of genetically-modified cassava wherever *Manihot* diversity is present. An alternative strategy could be based on a detailed understanding of gene flow in this species [1].

Crop scientists recognize that farmer practices have implications for gene flow; but rather than studying these practices directly, they have opted to make inferences on them based on genetic data [8], [15]–[16]. This is a sensible approach, perhaps the only one possible, when the focus is on historic populations, but not when seed management can be observed directly. Genetic data can be used confidently to test seed management's effects on gene flow and frequencies, but this requires sensible hypotheses that both recognize and understand the numerous practices involved.

It is generally taken for granted that seed management in traditional farming systems is well understood and amply documented. Our knowledge derives, in fact, from a handful of case studies that are not representative of a crop, a region or a farming system. Common generalizations have little empirical support. It is widely believed, for instance, that traditional farmers generally maintain a portfolio of crop diversity, when in fact it is most common to grow a single variety [10], [17]. A similar misunderstanding, in the context of seed exchange, is that fields are sown using seed from a single, familiar but otherwise random source [9]. Analyses of seed management also are largely descriptive and seldom based on a quantitative analytical framework [16]. Thus, landrace management most often is characterized simply as more or less dynamic, seed exchange as more or less frequent or widespread, and landrace populations themselves as more or less open [4], [8].

Seed and pollen exchange are both essential for the dispersal and persistence of alleles in cross-pollinated crops; but in contrast to pollen, seed is long lived and can be exchanged across long distances. “Seed” exchange (i.e., exchange of planting materials, including stakes and tubers) has an even greater weight on the gene flow of vegetatively-propagated crops, since “true seed” (i.e., fertilized ovules) and hence pollen often play no role in either formal or informal “seed” systems. Yet, seed movement rarely figures in models of gene flow in crops. Current models of transgene dispersal, for instance, focus exclusively on pollen [18]. These models are well suited to industrialized agriculture, where improved seed is replaced every cropping cycle, but not to traditional agriculture where farmers maintain landraces on farm [19], [20]. Farmers exchange seed within their own communities but also introduce seed from other localities. Sometimes they replace this seed for their own but may also mix both. All of these practices can be construed as events in the life history of a crop and articulated into a demographic model to shed light on management's role in its population dynamics (see Methods).

## Results

### Seed replacement

A log-linear model tested the effect of seed type and the locality's elevation on seed replacement rates (*1 – p*) [21]. G-tests for goodness of fit revealed significant differences in replacement across seed types (*P*<0.01) and elevation (*P*<0.01) (Table 1A). Landrace seed is replaced at significantly lower rates (*1 – p* = 0.25) than improved varieties (0.35). Freeman-Tukey deviates (not shown) revealed nevertheless that these differences are present only at low and intermediate elevations. Altitudinal differences in seed replacement are not significant for improved varieties; but differences are evident for landraces, whose seed is replaced at the highest rate at high elevations (*1 – p* = 0.33) and the lowest at intermediate elevations (0.15). The latter is the lowest rate of replacement of all type-by-altitude combinations.

**Table 1:** Various seed-management rates for cassava in Cauca, Colombia.^1^

	Type of seed		
	Landraces	Improved	Total
A. Replacement by elevation (N = 655)			
High	0.33	0.34	0.33
Intermediate	0.15	0.33	0.19
Low	0.21	0.38	0.28
Total	0.25	0.35	0.28
G elevation effect	17.4* (4 df)		
G type effect	12.0 *(3 df)		
B. Diffusion by origin^2^ (N = 165)			
Local	0.78	0.65	0.75
Introduced	0.72	0.88	0.79
Total	0.77	0.72	0.76
G origin effect	1.7 (2 df)		
G type effect	1.8 (2 df)		
G complete independence	1.9 (4 df)		
C. Introduction by elevation (N = 170)			
High	0.12	0.27	0.15
Intermediate	0.26	0.79	0.46
Low	0.14	0.13	0.13
Total	0.15	0.35	0.21
G elevation effect	19.4* (4 df)		
G type effect	11.9* (3 df)		
D. Mixing by origin (N = 165)			
Local	0.44	0.32	0.41
Introduced	0.61	0.39	0.50
Total	0.47	0.35	0.43
G origin effect	1.0 (2 df)		
G type effect	1.6 (2 df)		
G complete independence	2.1 (4 df)		

Significance at the 0.05 level is indicated by *.

1. Expressed as a ratio (varying between 0 and 1), replacement rates imply that seed is not saved across cycles; diffusion rates entail the exchange of saved seed; introduction rates mean that seed is brought into a locality.

2. Seed is “local” if acquired from neighbors and “introduced” if acquired in another locality.

### Seed diffusion

None of the newly-acquired seed in the sample was purchased from a commercial source, and only 2% was obtained from a non-governmental organization or directly from the Center for Tropical Agriculture (CIAT). The rest was acquired from other farmers. A log linear model tested the effect of seed type and origin on diffusion rates, i.e., the probability that a seed lot is distributed to other farmers. The model revealed no differences across either seed types (*P* = 0.41) or origin (*P* = 0.43; Table 1B). Inspection of rates across categories suggested a possible interaction of the effects of seed-type and origin, with landraces diffused more than improved varieties when seed is local but less when it is introduced. However, the interaction is not significant; a test of complete independence could not be rejected (*P* = 0.75), although possibly due to a reduced sample size (see below).

A separate log-linear model tested the effect of elevation and seed type on diffusion rates. In contrast to seed replacement, no systematic differences in diffusion rates were found across elevations (*P* = 0.19; Table 2A); but controlling for elevation revealed the effect of seed type on seed diffusion. Diffusion is higher for landraces than for improved varieties: *q* = 0.92 and 0.84, respectively (*P*<0.01). In this case too, differences between seed types are present only at low and intermediate elevations. Despite the absence of systematic differences in diffusion across elevations, Freeman-Tukey deviates showed that improved seed at high elevations is diffused at higher rates than elsewhere, while landrace seed is diffused at lower rates.

**Table 2:** Seed diffusion rates for cassava in Cauca, Colombia.

	A. Diffusion by type(N = 633)			B. Diffusion by source^1^ (N = 691)			C. Diffusion by origin(N = 189)		
Elevation	landrace	improved	total	own	new	total	local	introduced	total
High	0.90	0.90	0.90	0.97	0.75	0.90	0.76	0.64	0.74
Intermediate	0.94	0.81	0.90	0.94	0.81	0.91	0.70	0.95	0.82
Low	0.95	0.83	0.92	0.93	0.72	0.87	0.66	0.80	0.68
Total	0.92	0.84	0.90	0.95	0.76	0.89	0.71	0.83	0.74
G elevation effect	6.1 (4 df)			5.4 (4 df)			6.7 (4 df)		
G origin effect							6.8 (3 df)		
G complete indep							26.4* (4 df)		
G source effect				50.6* (3 df)					
G type effect	14.8* (3 df)								

Significance at the 0.05 level is indicated by *.

1. Seed acquired during the current cycle is “new;” seed saved by the farmer from a previous cycle is his/her “own”.

A third model showed that diffusion depends significantly on whether seed has been saved across cycles (i.e., farmers' own seed) or acquired during the last cycle (i.e., new seed) (Table 2B). Farmers' own seed is diffused at higher rates than new seed: 0.95 vs. 0.76, respectively (*P*<0.001). Again, no systematic differences in diffusion across elevations were found (*P* = 0.25), but Freeman-Tukey deviates showed significant altitudinal differences for seed saved across cycles. Own seed is replaced at the highest rate at high elevations and the lowest rate at low elevations.

A final log-linear model found marginally insignificant differences in the diffusion of seed of local origin and introduced (*P* = 0.08; Table 2C). In this case too, no significant differences across elevations were found (*P* = 0.15). However, a test of complete independence is rejected (*P*<0.001). Freeman-Tukey deviates showed that introduced seed is diffused at higher rates at intermediate elevations than elsewhere. At intermediate elevations, introduced seed also is diffused at higher rates than seed acquired locally. No significant differences are present between other origin-by-elevation combinations.

### Seed introduction and mixing

Almost one fourth of all new seed in the sample, i.e., seed acquired in the last cycle, is introduced. A log-linear model was used to analyze differences in the rate of introduction across elevations and seed types (Table 1C). Significant seed-type (*P* <0.01) and altitudinal (*P* <0.05) effects are present. The rate of introduction of landraces is less than half that of improved varieties: *r* = 0.15 and 0.35, respectively. However, this is not true at every elevation; no differences between types are present at low elevations. Introduction of both landraces and improved varieties is highest at intermediate elevations. Improved varieties at intermediate elevations have the highest introduction rates of all type-by-altitude combinations. They are introduced at rates three times those of landraces at the same elevation and six times those of improved varieties at low elevations.

Farmers maintain an average 1.62 varieties of cassava. Landraces and improved varieties represent 71 and 23% of seed lots recorded in the sample, respectively. An additional 6% were identified by farmers as a mix of a landrace and an improved variety; but 43% were reported as having been grown mixed with other varieties at some point. The percentage is higher for landraces and introduced seed than for improved varieties and local seed, but differences are not significant (Table 1D). A log-linear model finds no significant effect of seed type (*P* = 0.45) or origin (*P* = 0.62) on mixing. A test of complete independence could not be rejected (*P* = 0.73).

### Population growth rates

Growth rates of several cassava populations were estimated based on the parameters described above [10], [19]. A growth rate equal to 1 would imply that the population's size is constant across cycles; a rate above/below 1 would imply that the population grows/declines in numbers. The estimated rate of growth of improved varieties across Cauca is *λ* = 3.82, while landraces grow at a slightly lower rate: *λ* = 3.72. Growth rates are highest at mid elevations for both cassava landraces (*λ* = 4.06) and improved varieties (*λ* = 4.02). But while landrace populations grow the least at high elevations (*λ* = 3.61), improved varieties experience their lowest growth at low elevations (*λ* = 3.35). Finally, the growth rates of improved varieties at high elevations and landraces at low elevations are 3.76 and 3.90, respectively.

## Discussion

Biotechnology is expected to have a greater impact than classical crop breeding on vegetatively-propagated crops [22]–[24]. Genetically-modified (GM) cassava could spread widely across developing areas where farmers still rely on landraces. Surprisingly, discussions on biosafety have largely neglected the implications of vegetative propagation for current strategies to contain transgenes. In 1996, shortly before the commercial release of GM maize in the United States, experts took for granted that this germplasm would spread quickly, carried by farm workers across international borders from areas of industrialized agriculture into maize's center of diversity in Mexico [25]. Farming practices would then facilitate the diffusion and introgression of transgenes into maize landraces. Indeed, transgenes were detected in Mexican maize landraces in 2001 [26], and they had spread widely by 2002 [19].

Cassava's biology and mode of propagation is believed to reduce the risk of unintentional transgene spread and establishment vis-à-vis grain crops [27]. Flowering times, genetic compatibility factors, low fecundity and dormancy all seem to limit gene flow in cassava. It has been suggested that while appropriate isolation distances would reduce the risk of out-crossing, cassava's clonal propagation and herbicides (to remove volunteers) could prevent novel traits from being passed on if out-crossing were to occur [27]. Confined handling and transport of cassava stakes also seems less challenging than that of grains. Cassava's multiplication potential via stakes is only 1/2,250 that of maize via seed [14]; moreover, stakes lose viability quickly, have no dormancy, are less likely to be lost, and are easily prevented from establishing and surviving in the environment [14], [27]. The consequences of containment failures, it is argued, are thus of less concern with cassava than with other crops.

Although little is known about GM germplasm's possible fate after its release [14], on-farm management of cassava seems to be extremely conservative vis-à-vis maize. Cassava seed exchange among indigenous Guyanian farmers, for instance, is quite formal and largely restricted to close kin and neighbors [6]. Exchanges outside family, village or ethnic boundaries are reportedly very occasional, suggesting that informal seed systems are surprisingly closed. Nevertheless, occasional exchanges across hundreds of miles have been reported. Moreover, indigenous farmers are known to actively incorporate seedlings from soil seed banks, which presumably facilitates unintentional gene flow across successive occupants' stocks and into wild populations [2]. But to what extent are these practices representative of cassava's center of diversity? We are unaware of systematic analyses of cassava management across any region. In what follows we analyze cassava's management and population dynamics across Cauca, comparing them to those of maize in Mexico.

### Open populations and dynamic management

Our results reveal both differences and similarities in the management of cassava and maize. Average replacement rates for cassava in Cauca are only slightly lower than those estimated for maize across Mexico (1 *– p* = 0.28 and 0.32, respectively) [10]. But when the focus is on improved varieties, it is clear that cassava is replaced at much lower rates than maize (1 *– p* = 0.35 and 0.79, respectively) (Table 1A). This is not surprising since improved maize consists mostly of hybrid varieties, whose vigor decreases rapidly after the first cycle, while improved cassava can be maintained indefinitely via cloning. Observed rates suggest nevertheless that improved cassava is replaced every three years in average—i.e., a longer interval than for improved maize but shorter than for cassava landraces. Not surprisingly, improved cassava is introduced at much lower rates than improved maize (*r* = 0.35 vs. 0.76, respectively) (Table 1C).

The distribution of agricultural systems where maize is grown and their reliance on the formal seed system explains considerable differences in the management of improved varieties across regions and elevations in Mexico [10], [19]. Significantly, replacement rates for improved cassava vary little across elevations (1 *– p* = 0.33 *–* 0.38), but large differences in the rate of introduction are observed (*r* = 0.13 *–* 0.79). But these differences cannot be attributed to a particular agricultural system or its reliance on the formal seed system. Corpoica, Colombia's National Agricultural Research center, had a central role in the improvement and distribution of cassava germplasm in the past, but its presence in Cauca is currently negligible [20]. Most introduced cassava in the study region is acquired from farmers in neighboring localities. This exchange of germplasm presumably is initiated by request from a farmer in need of seed [5]. Not surprisingly, seed-diffusion rates are 7.5 times higher for improved cassava than improved maize (*q* = 0.84 and 0.11, respectively) (Table 2A). In sum, differences in the management of improved germplasm are clearly associated with these crops' biology but also due to institutional factors, e.g., the current absence of a well-developed formal seed system in Cauca.

Comparing the management of landraces is more revealing because farmers breed, maintain and exchange these varieties exclusively through informal systems. Cassava and maize landraces are replaced at nearly the same average rates (*1 – p* = 0.25 and 0.24, respectively). Across elevations, replacement rates range from 0.21 to 0.33 for cassava and 0.23 to 0.31 for maize. The source of this variation has not been sufficiently explained. Some analysts attribute altitudinal differences to environmental gradients: conditions at low altitudes are said to promote a more dynamic management of landrace populations [4]. However, there is no correlation between elevation and replacement rates in cassava (Table 1A) or in maize [19]. Interestingly, intermediate elevations exhibit the lowest replacement rates for cassava but the highest for maize. Similarities in replacement rates are nevertheless surprising given these crops' contrasting biology, but average introduction rates differ more markedly: cassava landraces are introduced at rates three times higher than maize landraces (Table 1C). Average diffusion rates also differ strikingly across crops (*q* = 0.92 for cassava and 0.20 for maize landraces). Across elevations, diffusion rates range from 0.90 to 0.95 for cassava (Table 2A) and from 0.15 to 0.22 for maize [19].

As with improved varieties, it is difficult to attribute particular aspects of landrace management to any single factor; but identifying the causes of farmer practices is beyond our present intent. Our estimates can be used nevertheless to describe cassava's population dynamics. Estimated diffusion and introduction rates suggest, for instance, that cassava populations are remarkably more dynamic (*q*) and open (*r*) than maize. A much more detailed exploration of these dynamics is yet possible.

### Diversity and non-local seed

Conceptions on non-local germplasm's impact on local diversity differ markedly across crops. Non-local seed is considered an important source of diversity for cassava but a threat to local maize diversity [3], [5]–[6]. Indeed, seed that is introduced into a locality can displace local germplasm when crop populations are of constant size and unstructured, i.e., when all seed is managed indistinctly [10]. The first of these conditions can occur when physical space is limited within individual farms; and its implications for diversity are clearest for an out-crossing species such as maize, whose racial ideotypes must be maintained through cross-pollination of relatively large populations [3]. In contrast, rare cassava genotypes can be preserved through asexual reproduction; and farmers apparently maintain as few as one or two mounds per farm [5]. But given that the number of mounds per farm remains finite, the size of cassava populations also can be considered constant.

Seed introduced into a locality can be compared to immigrants in a population. If “immigrant” seed lots and their descendants are construed as a distinct subpopulation, their abundance in the metapopulation can be stated as a function of seed management [10]. When seed is managed indistinctly after it is introduced into a locality—i.e., when introduced seed is replaced and diffused at the same rates as local seed—the proportion of non-local seed (i.e., immigrants and their descendants) depends entirely on the rate of introduction [10]. When seed is introduced only once, the relative abundance of non-local germplasm remains constant thereafter, but it grows with every cycle when introductions are continuous. If these conditions applied in Cauca, the growth rate of non-local cassava populations (*λ_nl_*) would be 0.21 points higher than that of local seed (*λ_l_*) (i.e., *λ_nl_* = *λ_l_*+0.21) or equal to the average rate of introduction (Table 1C). At intermediate elevations, where introduction rates are highest, the differential would be 0.46 points. The implication would be that cassava populations in Cauca consist almost entirely of seed introduced during the last few years. This need not be the case, however, if the second condition described above does not apply to Cauca, e.g., because introduced seed is replaced at higher rates or diffused at lower rates than local seed. In Mexico, introduced maize landraces and improved varieties are diffused at significantly lower rates than local seed (*q* = 0.13 and 0.23, respectively) [10]. Cassava shows a strikingly different pattern. Although differences observed are only marginally significant, introduced cassava tends to diffuse at higher rates than local germplasm. And these rates are extraordinary compared to those of maize. At intermediate elevations, for instance, 95% of introduced seed is diffused after its first cycle (i.e., *q* = 0.95).

The prevalence of non-local germplasm also depends on seed replacement; e.g., non-local populations will not expand if introduced seed is constantly discarded and replaced. Introduced maize landraces, for instance, are replaced as much as improved varieties [10]. This means that introduced maize is both replaced more and diffused less than local maize, thus requiring a constant influx of introduced seed for non-local populations to survive. The high replacement rate of maize suggests that seed is introduced for testing but found wanting and discarded, which could apply to cassava too. We were unable to estimate replacement rates for introduced cassava due to lack of sufficient data. Non-local cassava landrace populations nevertheless are bound to grow much faster than maize even if they are replaced at equally high rates, given that their introduction rates are three times higher. In fact, estimates for maize suggest that, in contrast to cassava, local and non-local populations grow at virtually the same rate (*λ_nl_*–*λ_l_*<0.001 in regions where traditional agriculture dominates) [10]. It seems certain thus that the share of non-local germplasm is much higher for cassava landraces than for maize.

Introduced cassava is diffused at similar rates whether it is a landrace or an improved variety; but the latter are introduced at higher rates (Table 1C). Whether a landrace or an improved variety, any germplasm should be considered local after it has been planted for more than one generation [3]. The median age of seed lots is higher for cassava than for maize (i.e., 5 and 3 years, respectively). But no seed over 20 years old was recorded in Cauca, while 18% of maize seed lots in southeast Mexico are at least 25 years old—i.e., old enough to be bequeathed across generations. We would need to consider differences in replacement rates across seed types and farms before concluding that most cassava in Cauca has been introduced in the last generation. What our results say unequivocally is that a greater proportion of improved cassava than landraces has been introduced recently. Results also suggest that improved cassava populations are growing faster than landraces, which could mean that the latter are being displaced (see below).

Indeed, when a metapopulation's size is fixed, the subpopulation that grows fastest eventually displaces the rest, assuming that growth rates are constant [10]. In some cases, however, growth might be inversely associated with a subpopulation's abundance—a process that might favor the spread of newly introduced germplasm but stop short of displacing local stocks. Density-dependence could also prevent dwindling populations from disappearing altogether, as reported in Guyana, where farmers rarely discard cassava varieties no longer favored by others [5]. In Mexico, newly introduced maize grows faster than all other subpopulations, but this growth is not long-lasting [10]. Our estimates suggest that Cauca farmers exchange cassava across localities and then distribute this germplasm among neighbors at rates much higher than Mexican maize; yet, local cassava is not replaced at higher rates than maize. It is possible, thus, that density-dependence is constraining the growth of introduced cassava populations and so seed exchange could actually be increasing the crop's diversity within individual localities. Does this mean that cassava farmers are hoarding diversity?

According to the literature, varietal richness is much higher for cassava than other crops, reaching up to 76 varieties per locality [5], [17]. This richness could also be associated with cassava's mixed reproductive system [6], [23], [28]. However, richness estimates at the locality level can be misguiding (see Methods). When the focus is on individual farms, cassava's diversity does not stand out from other crops [17]. Our data shows that the average number of varieties maintained by cassava farmers in Cauca is only slightly higher than the number reported for maize farmers in south-central Mexico (1.62 and 1.44, respectively) [10]. Is it possible then that farmers are not registering the diversity introduced via seed exchange or that they are losing it inadvertently? Guyanian farmers reportedly recall the origin of every variety they acquire; but farmers might not always recognize differences between newly acquired seed and their own, leading them to mix genotypically distinct germplasm [5]. This “confusion” could prevent an increase in varietal richness but simultaneously promote intra-varietal diversity (see below). Indeed, exchanging large amounts of germplasm across localities might be a way of offsetting intra-varietal loss of diversity due to management (e.g., through seed selection) [5], particularly if this loss is greater in clonally-propagated plants than in sexually-reproduced crops such as maize [10].

### Improved varieties vs. landraces

Scholars have long associated the spread of improved varieties with the loss of crop diversity; yet, the evidence remains inconclusive [29]. Moreover, improved varieties have not spread across developing areas as widely as expected, and recent surveys suggest that farmers still maintain considerable landrace diversity [17]. Sales records of improved varieties are the most common measure of use, but sales can underestimate the abundance of improved germplasm in the fields because this seed too can be saved and “recycled.” Still, it is possible to estimate changes in the abundance of improved varieties by analyzing the growth rate of their populations within the crop's metapopulation.

Sales records suggest that introduced improved maize was more common in Mexico during the mid nineties than at present—a process associated with the expansion of irrigated agriculture outside maize's center of diversity, that is, with an expanding metapopulation. Clearly, this does not imply that improved populations displaced maize landraces, which might have expanded too despite exhibiting lower growth rates. Subpopulations exhibiting subpar growth can expand when the metapopulation increases in size [10]. But the area in maize has decreased gradually across Mexico since then. Growth rate estimates suggest nevertheless that maize landrace populations are stable (*λ* = 1.03); but improved varieties would dwindle (*λ* = 0.33) due to their high replacement and low diffusion if not infused continuously with new germplasm (i.e., through formal seed systems). In sum, there is no clear evidence that improved varieties have displaced maize landraces in Mexico [10].

Cassava's population dynamics are very different: current infusions of improved germplasm through formal seed systems are noticeably rare; but existent populations are diffused at rates seven times higher than improved maize, and their rate of survival (*p*) also is twice as high. In the cassava metapopulation, improved germplasm exhibit higher replacement rates than landraces but also higher introduction rates. The first fact reduces a possible growth differential between these populations, but the second one increases it. According to our estimates, the growth rate of improved varieties (*λ_iv_*) is 0.10 points higher than that of landraces (*λ_lr_*) (i.e., *λ_iv_* – *λ_lr_* = 0.10). Surprisingly, both populations seem to grow at exceedingly high rates (i.e., 3.82 and 3.72, respectively). Several factors can explain these results.

An expansion of cassava agriculture could be the main reason behind growing populations in Cauca. “Massive exchanges” of large amounts of planting material seem to take place both when new farms are established and when farmers sow large fields [5]. Survey data show that the area sown to cassava in Cauca increased 30% in 2010 after several years of contracting. At the same time, our growth estimates assume that seed from each source becomes a separate seed lot, i.e., that seed lots are grown and maintained as a distinct type. But if planting material is in short supply, farmers may be combining seed from several sources to sow a single field (or form a single seed lot). As discussed earlier, farmers may be mixing genotypically distinct but phenotypically similar seed (i.e., seed of the same named variety) into a single seed lot, inadvertently increasing its diversity. They may also be mixing different varieties on purpose.

Mexican maize farmers are known to mix varieties (particularly landraces and improved varieties) with the intention of hybridizing them, i.e., creolizing improved varieties or improving local varieties [30]. Growing mixed stands of cassava is a common practice in Cauca (Table 1D); but farmers' intentions are not obvious given cassava's clonal propagation. Amerindian farmers are known to incorporate cassava seedlings into their stocks of clones, favoring large-sized, heterozygous individuals—a practice that increases genotypic diversity or might even generate new varieties [7], [23]. However, it is uncertain whether this is an intended or completely inadvertent outcome [5], [6]. Moreover, there are few indications that the practice is widespread among cassava farmers. Cauca farmers reportedly incorporate volunteers opportunistically, and significantly, 6% of seed lots in the region are considered hybrids. However, there are no reports of farmers purposely hybridizing varieties. Growing mixed stands could also be a strategy to mitigate the risks posed by a complex and changing environment. Significant variability in the response of different varieties to nutrient availability and in resistance to drought and pests suggests that a mixed stand could help farmers stabilize yields and secure a harvest [31].

A clearer understanding of these issues is needed before mixing can be modeled as part of cassava's demography. This gap in our knowledge notwithstanding, several conclusions can be drawn. Cassava populations' surprising growth rates are due to the high introduction and diffusion rates of landraces (vis-à-vis maize) but to high diffusion and survival rates in the case of improved varieties. Far from being the random process implied by current models [9], these differences could reflect the diffusion of technological innovations, the expansion of agriculture, or multiple other factors influencing farmer decisions. To what extent these social process have played a role in Cauca is a complex question, particularly when we consider the interdependence of introduction, replacement and diffusion rates. The higher replacement of improved cassava than of landraces, for instance, might be tied to the frequent introduction of ill-suited germplasm. Alternatively, farmers might be introducing new seed to replace local germplasm that has (or seems to have) decayed [32]. Observed differences in the dynamics of cassava populations across Cauca can shed light on alternative possibilities.

According to our estimates, improved cassava populations exhibit lower growth than landraces at low elevations but higher growth at high elevations. Since landraces at high elevations are replaced and diffused at the same rates as improved varieties, the latter's advantage is due exclusively to introductions, which occur at over twice the rate for landraces (Table 1). This suggests that new improved germplasm is replacing not only older improved varieties but possibly also landraces. Improved cassava also is introduced at much higher rates than landraces at mid elevations, but here the latter are saved and diffused at higher rates, so growth rates are similar for both subpopulations. Thus, at mid elevations, improved cassava might be introduced mostly to replace its own populations. At low elevations, landraces also are saved and diffused at higher rates than improved varieties, but both groups are introduced at the same low rate, suggesting that local landraces could be regaining ground against improved varieties.

### Implications for biosafety

Analysts described the introgression of maize transgenes into Mexican landraces—and their presumed disappearance—as both unsurprising and inevitable [33]. In fact, transgenes have not disappeared but dispersed widely, and their sources and mechanisms of dispersal remain controversial [19]. It is now clear that pollen cannot explain transgene dispersal at a geographical level; but neither can farmer practices alone explain the abundance and distribution of transgenes across Mexico. Predictive models will need to consider germplasm's simultaneous flow through formal and informal seed systems, as well as the movement of grain through markets [19].

Cassava's case is different to the extent that its seed is not traded as food or feed, but there are also similarities with maize. Cassava's biology and vegetative propagation might facilitate the confinement of field trials, as expected [27], but simultaneously promote the diffusion of GM germplasm once it is released. The dynamic exchange of seed observed in Cauca could grant local farmers faster access to biotechnological innovations than their counterparts in Mexico despite the lack of a well-developed seed system. Other traditional-farming practices (i.e., mixing seed and incorporating seedlings into seed stocks) could also allow these farmers to transfer useful transgenes into locally-adapted landraces. These same practices might also allow GM varieties to spread unrestrictedly, whether they have been released intentionally or accidentally. Our findings suggest that GM cassava is unlikely to displace landraces or compromise their diversity, but other hazards cannot be ignored. Transgenes that increase cassava's qualities as an industrial crop or as feed, for instance, could compromise food safety if farmers cannot recognize them. This could be the case of transgenes coding for industrial proteins such as pharmaceuticals. Food crops are ideal hosts for the synthesis of industrial proteins in terms of practicality, economy, ease of storage and distribution, but their use also entails poorly known risks [34]–[36].

We should expect farmers to manage GM cassava like any other improved germplasm. That improved cassava is commonly saved across cycles, unlike hybrid maize, increases the likelihood of gene flow across fields and into seed banks. Given that farmers often prefer growing cassava in rented land, seed banks could facilitate the inadvertent diffusion of transgenes across households. Deliberate exchange of seed is itself surprisingly frequent, and exchanges across localities are much more often for improved cassava than for landraces. This constant introduction would allow GM cassava to spread across localities rapidly, in contrast to maize, whose improved germplasm very rarely spreads. The diffusion and subsequent incorporation of GM germplasm into seed banks could obstruct the tracking and eradication of deleterious transgenes in cassava to a greater extent than in maize. Attempts to regulate these and other traditional farming practices to reduce such risks could compromise the adaptive potential of cassava's populations and at the same time prove ineffectual [37].

## Methods

Our methods—i.e., model, data collection and analyses—and definitions follow the literature [10], [19]. For expediency, improved varieties and landraces are treated here as clearly delimited and mutually exclusive categories defined by their breeding history [3], [38]. Improved varieties, as opposed to landraces, consist of germplasm generated by a formal breeding program [3]. In practice, landraces are vaguely-circumscribed taxonomic units, and hence their alternative designation as “named varieties.” More precisely, a landrace is defined as the group of seed lots considered by farmers as belonging to the same type and thus given the same name; a seed lot is the set of propagules of a specific type selected by a farmer and sown during a cropping season to reproduce that particular type [3]. Since the same landrace can be given different names, the number of named varieties registered in an oral survey can overestimate actual varietal richness. At the same time, these estimates as well as those based on sample collections depend on the intensity of sampling. Clearly, none of these problems arise at the level of the individual farm. Farmers manage crop diversity by acting upon individual seed lots: *e.g.,* replacing one variety for another implies discarding one seed lot and taking up another; introducing a variety into a locality and diffusing it entails acquiring a seed lot from a non-local source and exchanging (i.e., distributing) it among fellow farmers, thereby producing new seed lots. Likewise, mixing varieties means growing two seed lots in a mixed stand.

### Model

Consider a closed population *N* consisting of *N_t_* seed lots at time *t*. At the end of the period, seed lots are saved with probability *p* and diffused with probability *q* from one farmer to *C* others. These new seed lots become part of the *t+1* population along surviving lots, such that *N_t+1_* = *(p*+*qC) N_t_*. More generally, assuming constant survival and diffusion probabilities over time, population size at *t* is given by *N_t_* = *(p*+*qC)^t^ N_0_* = *λ^t^ N_0_*, where *λ* is the population's expected growth rate. The population grows (*i.e.*, *λ*>1) if seed diffusion offsets seed loss or replacement. If survival and diffusion probabilities are the same for seed lots in *N*, *λ* is the growth rate of both *N* and every seed line in it. But even if *λ*>1, specific seed lots or seed lines can become extinct unless there is a perfect negative correlation between seed survival and diffusion.

If there is a one-time introduction of non-local seed into the population at *t* = *τ*, such that *N* incorporates *rN_τ-1_* introduced seed lots along with saved and locally diffused seed, then *N_τ_* = *(p*+*qC*+*r)N_τ-1_*. The number and proportion of introduced seed lots are, respectively, *N_I,τ_* = *rN_τ-1_* and *n_I,τ_* = *N_I,τ_ /N_τ_* = *r/(p*+*qC*+*r).* Assuming that introduced lots are saved and diffused at the same rate as the local lots, the population grows at a rate of *λ* = *p*+*qC* after *τ*, so that *N_t_* = *λ^ t-τ^ N_τ_.* Thus, the population at *t* consists of surviving lines (*i.e*., original lots plus copies) of the mixed-origin *τ* population, and the proportion of non-local seed (*i.e.*, introduced lots plus copies) is constant. If introductions are continuous, the rate of introduction (*r*) becomes part of the population growth rate: *N_t_* = *(p*+*qC*+*r)^t^ N_0_* = *λ^t^N_0_*. Since the local subpopulation grows at the rate of *λ_L_* = *p+qC*<*λ*, the proportion of local lots in the population decreases continuously: *n_L,t_* = *N_L,t_ / N_t_* = *(1 – r/ λ)^t^ n_L,0_*. At carrying capacity, *λ* = *1* and *λ_L_<1*, so the number of local lots drops exponentially until they are completely replaced by introduced seed.

The dynamics of distinct seed types can be analyzed by letting *N_I_* and *N_J_* represent separate subpopulations of *N*. If all rates are homogeneous across subpopulations, then both *N_I_* and *N_J_* grow at the rate of *λ* = *p*+*qC*+*r*. If rates differ, *N_I,t_* = *(p_I_*+*q_I_C*+*r_I_)^t^ N_I,0_* (and likewise for *N_J,t_*). Interactions between subpopulations can be made explicit by decomposing diffusion and introduction rates: *q_I,0_* = *q_II_*+*q_IJ_ n_JI,0_* and *r_I,0_* = *r_II_*+*r_IJ_ n_JI,0_*, where *q_II_* and *q_IJ_* are, respectively, diffusion rates of seed lots in *N_I_* with respect to itself and w.r.t. *N_J_* (and likewise for *r_II_* and *r_IJ_*), and *n_JI,t_* = *N_J,t_/N_I,t_* represents relative abundance at time *t*. Substituting and regrouping terms, *N_I,t_* = *λ_I,t-1_ N_I,t-1_* = *(λ_II_*+*s_IJ_ n_JI,t-1_)N_I,t-1_*, where *λ_II_* = *p_I_*+*q_II_C*+*r_II_* and *s_IJ_* = *q_IJ_C*+*r_IJ_* represent subpopulation *N_I_*'s intrinsic growth and its interaction with subpopulation *N_J_*, *e.g*., seed replacement within *N_I_* and replacement of variety *N_J_* by *N_I_*. Growth of *N_I_* is thus a function of *n_JI,t_*, whose rate of change is itself the ratio of *N_I_*'s and *N_J_*'s growth rates: *n_JI,t_* = *(λ_J,t-1_/λ_I,t-1_)n_JI,t-1_*.

Inspection of the previous equation reveals two possible stable equilibria *n_JI_*: either growth rates balance out and subpopulations coexist, or one subpopulation prevails and the other becomes extinct; *i.e.*, *n_JI_* = 0 or ∞. When *λ_JJ_* = *λ_II_* and *s_JI_* = *s_IJ_*, *n_JI_* converges to 1. If rates differ across types, subpopulations coexist as long as there is a strictly positive solution for *n_JI_* in *λ_JJ_* – *λ_II_* = *s_IJ_n_JI_ – s_JI_n_JI_^-1^*; that is, as long as intrinsic growth differences are offset by replacement across populations. When differences are restricted to interaction terms (*i.e*., *λ_JJ_* = *λ_II_* and *s_IJ_ ≠ s_JI_*), there is an analytical solution: *n_JI_* = (*s_JI_* /*s_IJ_*)*^0.5^*. Subpopulations coexist whenever there is either some cross-replacement (*s_IJ_>s_J_* >0) or none at all (*s_IJ_* = *s_JI_* = 0) but not when replacement is one-sided (*s_IJ_>s_JI_* = 0).

### Data analysis

A survey of 275 farms across14 municipalities in the Department of Cauca, Colombia, conducted in early 2010, provided data on 719 cassava seed lots. The survey was based on a stratified-random sample to ensure that it is representative of cassava farmers in Cauca (but not necessarily of farmers in smaller areas within this region) [20]. Stratification across municipalities (based on the area sown to cassava) does not explain the role played by social and environmental factors within political divisions, but it reduces the variance of descriptive statistics, ensuring 95% confidence for estimates at the household level. Since households are the elementary sampling units, the effects of sample design are restricted to this level. That is, there are no additional effects (due to deviations from simple random sampling) at the seed-lot level since all seed lots owned by sample households were considered in the analysis; i.e. seed lots were censused. We used seed-lot data to estimate rates of seed replacement, diffusion, introduction and mixing. Rate differences across seed types were then determined through the analysis of three-way tables based upon log-linear models [21]. This analysis was not intended as an exhaustive breakdown of the causal factors involved in seed management but as a way of identifying differences in management across specific seed types (or populations) often addressed in the literature [16]. These include improved varieties and landraces, seed maintained at different elevations, and seed of different geographic origin [3]-[4], [10], [19]. The three altitudinal regions considered here are high (>1600 masl), intermediate (1200–1600 masl) and low (<1200 masl) elevations. The influence of cassava breeding programs and the nature of germplasm diffused through formal seed systems differ markedly across these regions [20]. Finally, rate differences observed across seed types were used to describe disparities in these populations' dynamics [10], [19].
